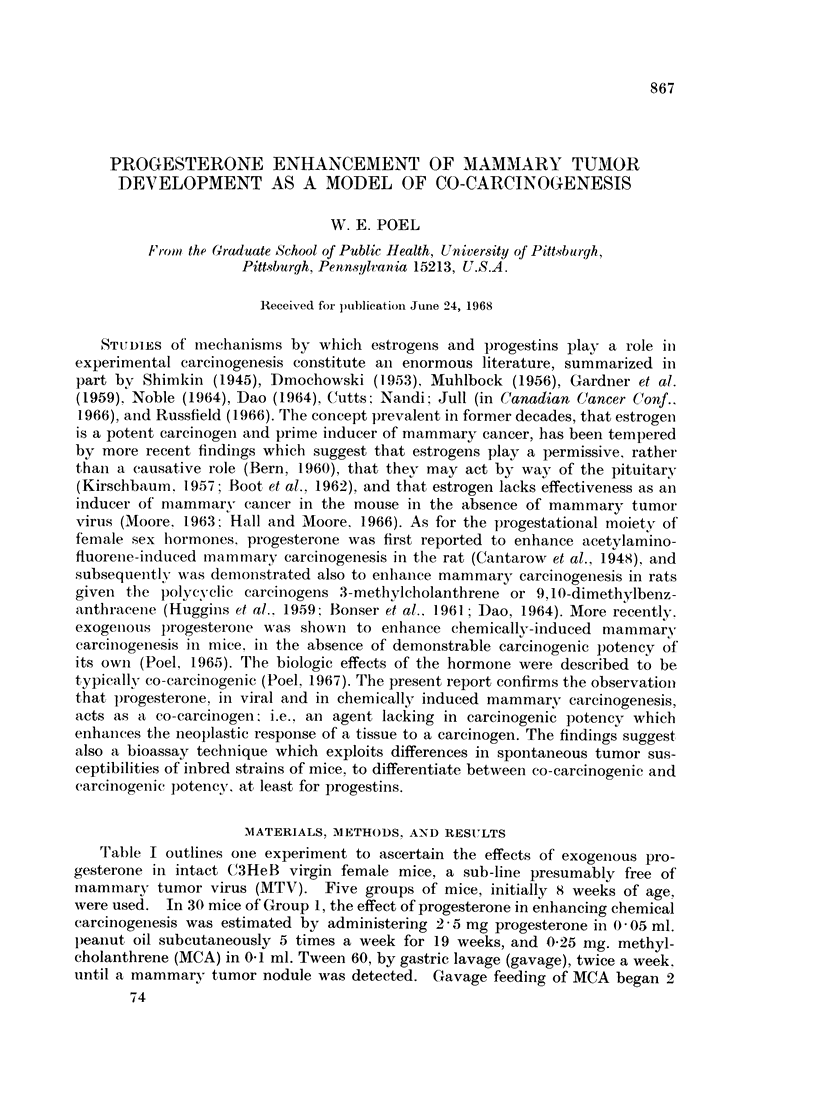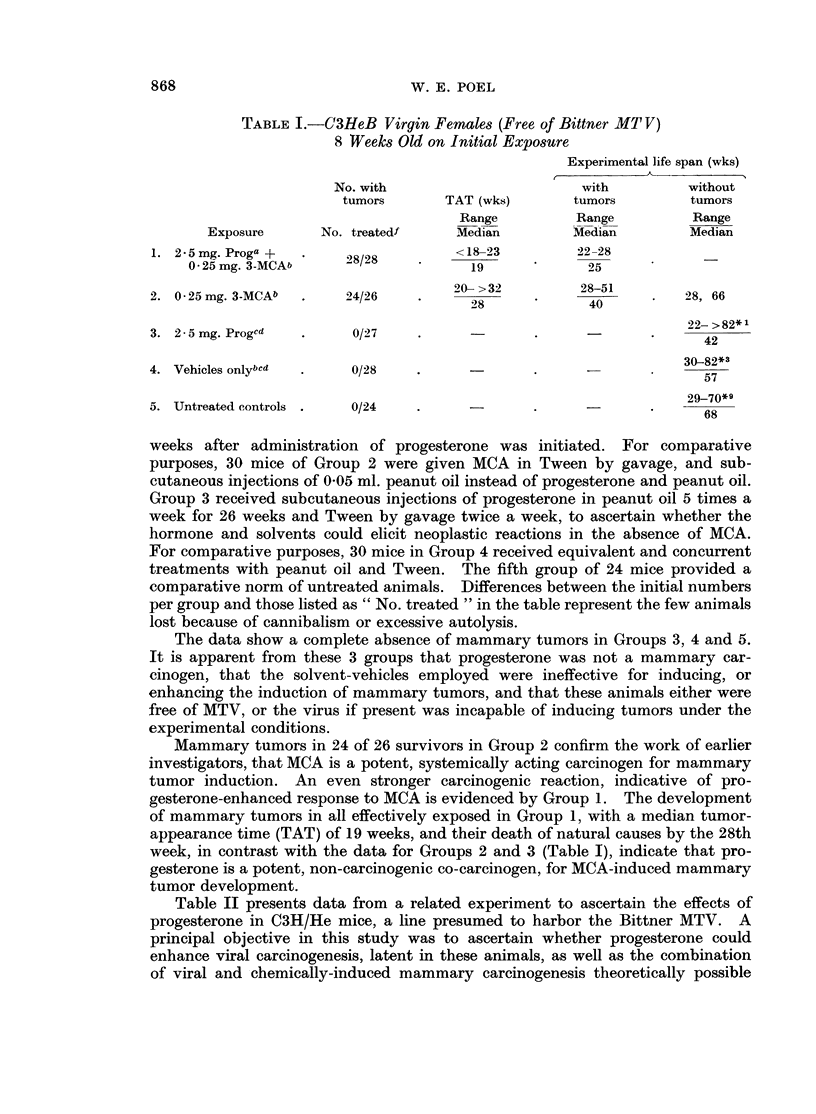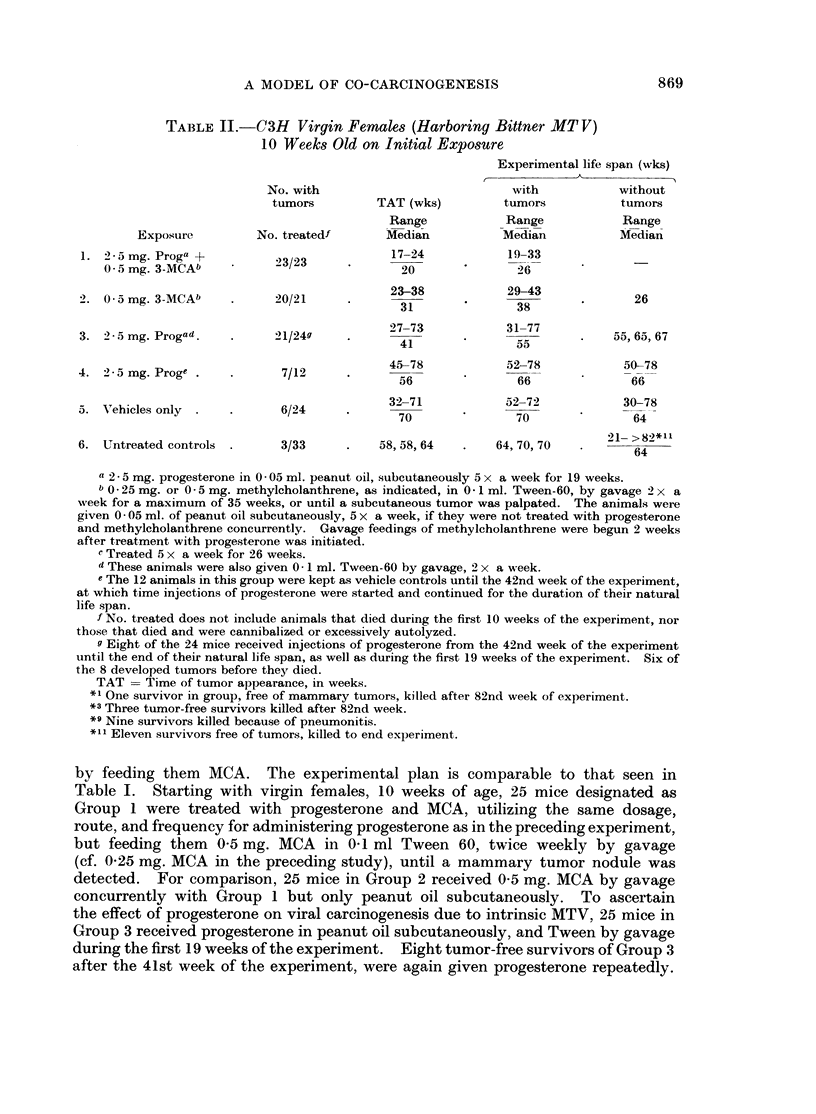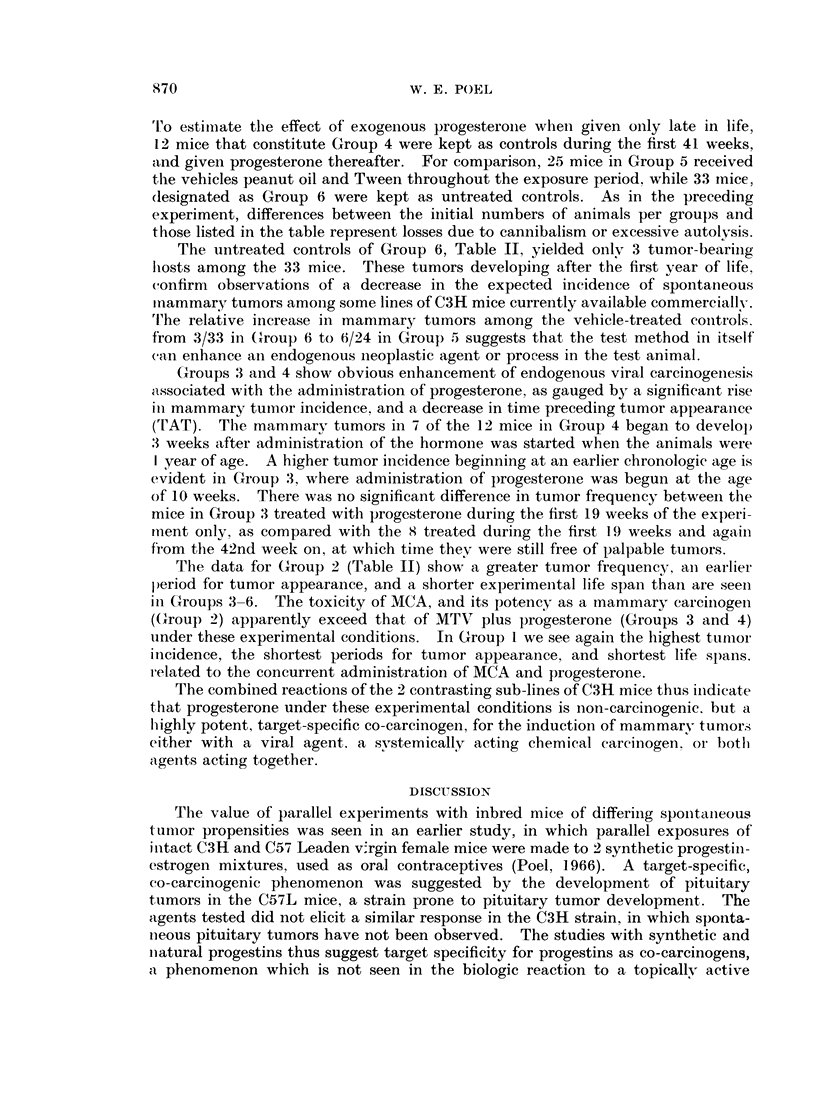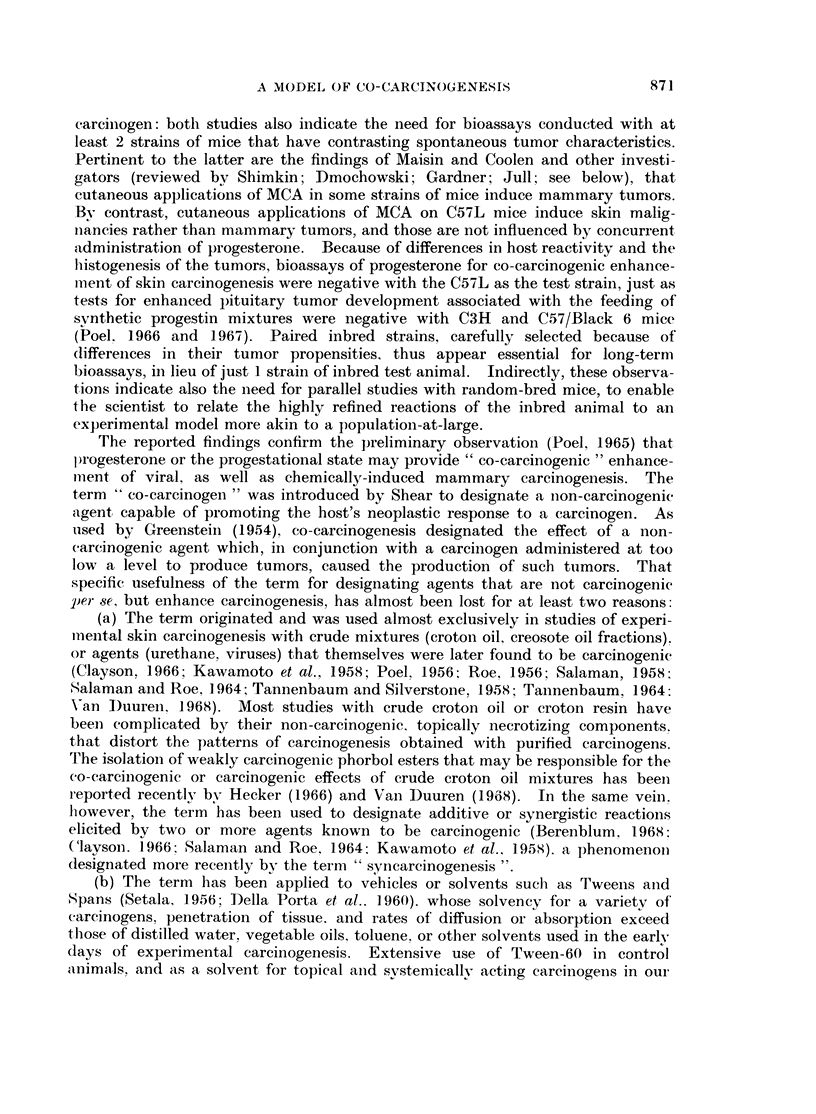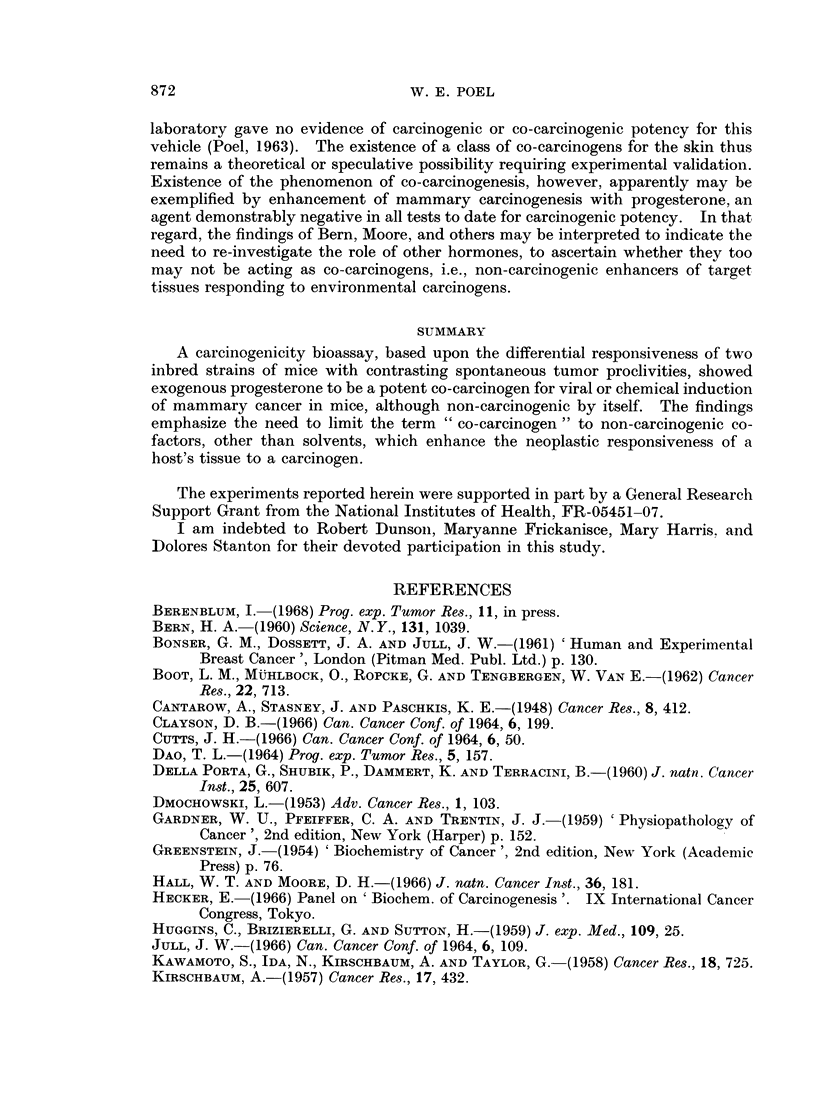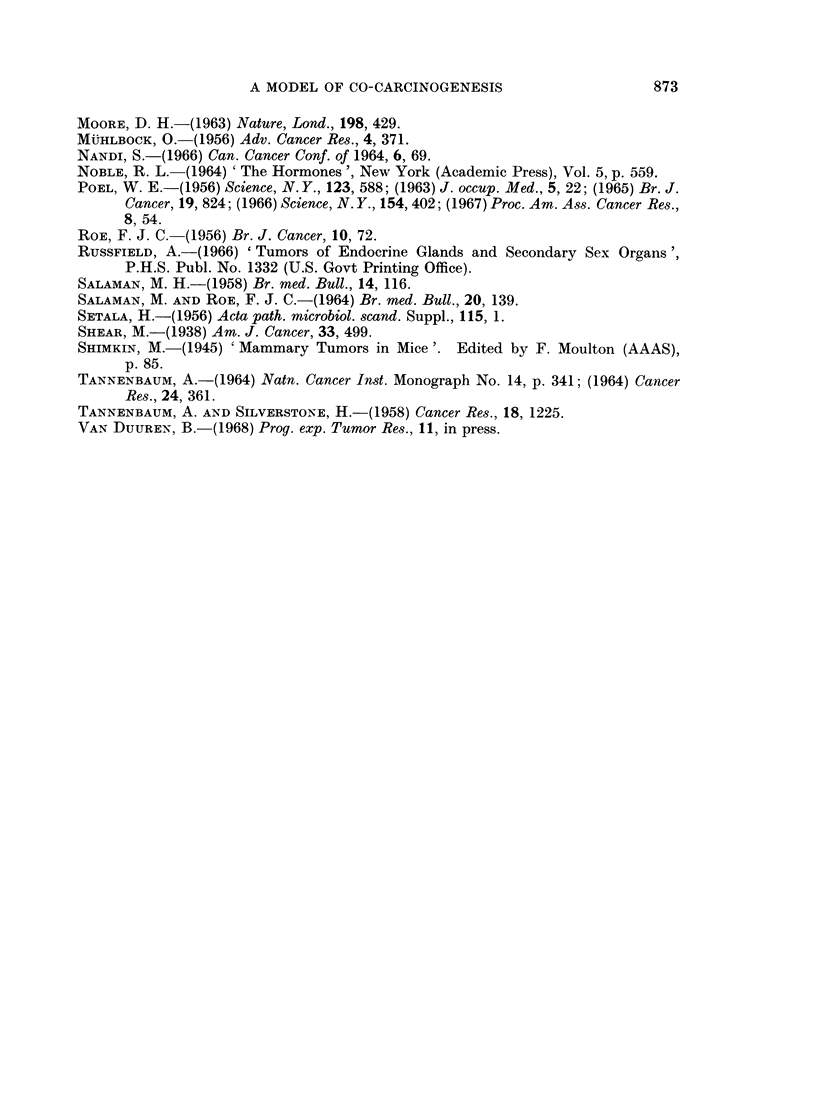# Progesterone enhancement of mammary tumor development as a model of co-carcinogenesis.

**DOI:** 10.1038/bjc.1968.102

**Published:** 1968-12

**Authors:** W. E. Poel


### PROGESTERONE ENHANCEMENT OF MAMMARY TUMOR DEVELOPMENT AS A MODEL OF CO-CARCINOGENESIS

W. E. POEL

*From the Graduate School of Public Health, University of Pittsburgh, Pittsburgh, Pennsylvania* 15213, *U.S.A.*

Received for publication June 24, 1968

STUDIES of mechanisms by which estrogens and progestins play a role in experimental carcinogenesis constitute an enormous literature, summarized in part by Shimkin (1945), Dmochowski (1953), Muhlbock (1956), Gardner *et al.* (1959), Noble (1964), Dao (1964), Cutts; Nandi; Jull (in *Canadian Cancer Conf.*, 1966), and Russfield (1966). The concept prevalent in former decades, that estrogen is a potent carcinogen and prime inducer of mammary cancer, has been tempered by more recent findings which suggest that estrogens play a permissive, rather than a causative role (Bern, 1960), that they may act by way of the pituitary (Kirschbaum, 1957; Boot *et al.*, 1962), and that estrogen lacks effectiveness as an inducer of mammary cancer in the mouse in the absence of mammary tumor virus (Moore, 1963; Hall and Moore, 1966). As for the progestational moiety of female sex hormones, progesterone was first reported to enhance acetylaminofluorene-induced mammary carcinogenesis in the rat (Cantarow *et al.*, 1948), and subsequently was demonstrated also to enhance mammary carcinogenesis in rats given the polycyclic carcinogens 3-methylcholanthrene or 9,10-dimethylbenzanthracene (Huggins *et al.*, 1959; Bonser *et al.*, 1961; Dao, 1964). More recently, exogenous progesterone was shown to enhance chemically-induced mammary carcinogenesis in mice, in the absence of demonstrable carcinogenic potency of its own (Poel, 1965). The biologic effects of the hormone were described to be typically co-carcinogenic (Poel, 1967). The present report confirms the observation that progesterone, in viral and in chemically induced mammary carcinogenesis, acts as a co-carcinogen; i.e., an agent lacking in carcinogenic potency which enhances the neoplastic response of a tissue to a carcinogen. The findings suggest also a bioassay technique which exploits differences in spontaneous tumor susceptibilities of inbred strains of mice, to differentiate between co-carcinogenic and carcinogenic potency, at least for progestins.

### MATERIALS, METHODS, AND RESULTS

Table I outlines one experiment to ascertain the effects of exogenous progesterone in intact C3HeB virgin female mice, a sub-line presumably free of mammary tumor virus (MTV). Five groups of mice, initially 8 weeks of age, were used. In 30 mice of Group 1, the effect of progesterone in enhancing chemical carcinogenesis was estimated by administering 2·5 mg progesterone in 0·05 ml. peanut oil subcutaneously 5 times a week for 19 weeks, and 0·25 mg. methylcholanthrene (MCA) in 0·1 ml. Tween 60, by gastric lavage (gavage), twice a week, until a mammary tumor nodule was detected. Gavage feeding of MCA began 2

TABLE I.—*C3HeB Virgin Females (Free of Bittner MTV) 8 Weeks Old on Initial Exposure*

| Exposure | No. with tumors / No. treated[f] | TAT (wks) Range / Median | Experimental life span (wks): with tumors Range / Median | Experimental life span (wks): without tumors Range / Median |
|---|---|---|---|---|
| 1. 2·5 mg. Prog[a] + 0·25 mg. 3-MCA[b] | 28/28 | <18–23 / 19 | 22–28 / 25 | — |
| 2. 0·25 mg. 3-MCA[b] | 24/26 | 20– >32 / 28 | 28–51 / 40 | 28, 66 |
| 3. 2·5 mg. Prog[cd] | 0/27 | — | — | 22– >82*[1] / 42 |
| 4. Vehicles only[bcd] | 0/28 | — | — | 30–82*[3] / 57 |
| 5. Untreated controls | 0/24 | — | — | 29–70*[9] / 68 |

weeks after administration of progesterone was initiated. For comparative purposes, 30 mice of Group 2 were given MCA in Tween by gavage, and subcutaneous injections of 0·05 ml. peanut oil instead of progesterone and peanut oil. Group 3 received subcutaneous injections of progesterone in peanut oil 5 times a week for 26 weeks and Tween by gavage twice a week, to ascertain whether the hormone and solvents could elicit neoplastic reactions in the absence of MCA. For comparative purposes, 30 mice in Group 4 received equivalent and concurrent treatments with peanut oil and Tween. The fifth group of 24 mice provided a comparative norm of untreated animals. Differences between the initial numbers per group and those listed as " No. treated " in the table represent the few animals lost because of cannibalism or excessive autolysis.

The data show a complete absence of mammary tumors in Groups 3, 4 and 5. It is apparent from these 3 groups that progesterone was not a mammary carcinogen, that the solvent-vehicles employed were ineffective for inducing, or enhancing the induction of mammary tumors, and that these animals either were free of MTV, or the virus if present was incapable of inducing tumors under the experimental conditions.

Mammary tumors in 24 of 26 survivors in Group 2 confirm the work of earlier investigators, that MCA is a potent, systemically acting carcinogen for mammary tumor induction. An even stronger carcinogenic reaction, indicative of progesterone-enhanced response to MCA is evidenced by Group 1. The development of mammary tumors in all effectively exposed in Group 1, with a median tumor-appearance time (TAT) of 19 weeks, and their death of natural causes by the 28th week, in contrast with the data for Groups 2 and 3 (Table I), indicate that progesterone is a potent, non-carcinogenic co-carcinogen, for MCA-induced mammary tumor development.

Table II presents data from a related experiment to ascertain the effects of progesterone in C3H/He mice, a line presumed to harbor the Bittner MTV. A principal objective in this study was to ascertain whether progesterone could enhance viral carcinogenesis, latent in these animals, as well as the combination of viral and chemically-induced mammary carcinogenesis theoretically possible

TABLE II.—*C3H Virgin Females (Harboring Bittner MTV) 10 Weeks Old on Initial Exposure*

| | Exposure | No. with tumors / No. treated[f] | TAT (wks) Range / Median | Experimental life span (wks) with tumors Range / Median | Experimental life span (wks) without tumors Range / Median |
|---|---|---|---|---|---|
| 1. | 2·5 mg. Prog[a] + 0·5 mg. 3-MCA[b] | 23/23 | 17–24 / 20 | 19–33 / 26 | — |
| 2. | 0·5 mg. 3-MCA[b] | 20/21 | 23–38 / 31 | 29–43 / 38 | 26 |
| 3. | 2·5 mg. Prog[ad]. | 21/24[g] | 27–73 / 41 | 31–77 / 55 | 55, 65, 67 |
| 4. | 2·5 mg. Prog[e] | 7/12 | 45–78 / 56 | 52–78 / 66 | 50–78 / 66 |
| 5. | Vehicles only | 6/24 | 32–71 / 70 | 52–72 / 70 | 30–78 / 64 |
| 6. | Untreated controls | 3/33 | 58, 58, 64 | 64, 70, 70 | 21– >82*11 / 64 |

[a] 2·5 mg. progesterone in 0·05 ml. peanut oil, subcutaneously 5 × a week for 19 weeks.
[b] 0·25 mg. or 0·5 mg. methylcholanthrene, as indicated, in 0·1 ml. Tween-60, by gavage 2 × a week for a maximum of 35 weeks, or until a subcutaneous tumor was palpated. The animals were given 0·05 ml. of peanut oil subcutaneously, 5 × a week, if they were not treated with progesterone and methylcholanthrene concurrently. Gavage feedings of methylcholanthrene were begun 2 weeks after treatment with progesterone was initiated.
[c] Treated 5 × a week for 26 weeks.
[d] These animals were also given 0·1 ml. Tween-60 by gavage, 2 × a week.
[e] The 12 animals in this group were kept as vehicle controls until the 42nd week of the experiment, at which time injections of progesterone were started and continued for the duration of their natural life span.
[f] No. treated does not include animals that died during the first 10 weeks of the experiment, nor those that died and were cannibalized or excessively autolyzed.
[g] Eight of the 24 mice received injections of progesterone from the 42nd week of the experiment until the end of their natural life span, as well as during the first 19 weeks of the experiment. Six of the 8 developed tumors before they died.
TAT = Time of tumor appearance, in weeks.
*1 One survivor in group, free of mammary tumors, killed after 82nd week of experiment.
*3 Three tumor-free survivors killed after 82nd week.
*9 Nine survivors killed because of pneumonitis.
*11 Eleven survivors free of tumors, killed to end experiment.

by feeding them MCA. The experimental plan is comparable to that seen in Table I. Starting with virgin females, 10 weeks of age, 25 mice designated as Group 1 were treated with progesterone and MCA, utilizing the same dosage, route, and frequency for administering progesterone as in the preceding experiment, but feeding them 0·5 mg. MCA in 0·1 ml Tween 60, twice weekly by gavage (cf. 0·25 mg. MCA in the preceding study), until a mammary tumor nodule was detected. For comparison, 25 mice in Group 2 received 0·5 mg. MCA by gavage concurrently with Group 1 but only peanut oil subcutaneously. To ascertain the effect of progesterone on viral carcinogenesis due to intrinsic MTV, 25 mice in Group 3 received progesterone in peanut oil subcutaneously, and Tween by gavage during the first 19 weeks of the experiment. Eight tumor-free survivors of Group 3 after the 41st week of the experiment, were again given progesterone repeatedly.

To estimate the effect of exogenous progesterone when given only late in life, 12 mice that constitute Group 4 were kept as controls during the first 41 weeks, and given progesterone thereafter. For comparison, 25 mice in Group 5 received the vehicles peanut oil and Tween throughout the exposure period, while 33 mice, designated as Group 6 were kept as untreated controls. As in the preceding experiment, differences between the initial numbers of animals per groups and those listed in the table represent losses due to cannibalism or excessive autolysis.

The untreated controls of Group 6, Table II, yielded only 3 tumor-bearing hosts among the 33 mice. These tumors developing after the first year of life, confirm observations of a decrease in the expected incidence of spontaneous mammary tumors among some lines of C3H mice currently available commercially. The relative increase in mammary tumors among the vehicle-treated controls, from 3/33 in Group 6 to 6/24 in Group 5 suggests that the test method in itself can enhance an endogenous neoplastic agent or process in the test animal.

Groups 3 and 4 show obvious enhancement of endogenous viral carcinogenesis associated with the administration of progesterone, as gauged by a significant rise in mammary tumor incidence, and a decrease in time preceding tumor appearance (TAT). The mammary tumors in 7 of the 12 mice in Group 4 began to develop 3 weeks after administration of the hormone was started when the animals were 1 year of age. A higher tumor incidence beginning at an earlier chronologic age is evident in Group 3, where administration of progesterone was begun at the age of 10 weeks. There was no significant difference in tumor frequency between the mice in Group 3 treated with progesterone during the first 19 weeks of the experiment only, as compared with the 8 treated during the first 19 weeks and again from the 42nd week on, at which time they were still free of palpable tumors.

The data for Group 2 (Table II) show a greater tumor frequency, an earlier period for tumor appearance, and a shorter experimental life span than are seen in Groups 3–6. The toxicity of MCA, and its potency as a mammary carcinogen (Group 2) apparently exceed that of MTV plus progesterone (Groups 3 and 4) under these experimental conditions. In Group 1 we see again the highest tumor incidence, the shortest periods for tumor appearance, and shortest life spans, related to the concurrent administration of MCA and progesterone.

The combined reactions of the 2 contrasting sub-lines of C3H mice thus indicate that progesterone under these experimental conditions is non-carcinogenic, but a highly potent, target-specific co-carcinogen, for the induction of mammary tumors either with a viral agent, a systemically acting chemical carcinogen, or both agents acting together.

### DISCUSSION

The value of parallel experiments with inbred mice of differing spontaneous tumor propensities was seen in an earlier study, in which parallel exposures of intact C3H and C57 Leaden virgin female mice were made to 2 synthetic progestin-estrogen mixtures, used as oral contraceptives (Poel, 1966). A target-specific, co-carcinogenic phenomenon was suggested by the development of pituitary tumors in the C57L mice, a strain prone to pituitary tumor development. The agents tested did not elicit a similar response in the C3H strain, in which spontaneous pituitary tumors have not been observed. The studies with synthetic and natural progestins thus suggest target specificity for progestins as co-carcinogens, a phenomenon which is not seen in the biologic reaction to a topically active carcinogen: both studies also indicate the need for bioassays conducted with at least 2 strains of mice that have contrasting spontaneous tumor characteristics. Pertinent to the latter are the findings of Maisin and Coolen and other investigators (reviewed by Shimkin; Dmochowski; Gardner; Jull; see below), that cutaneous applications of MCA in some strains of mice induce mammary tumors. By contrast, cutaneous applications of MCA on C57L mice induce skin malignancies rather than mammary tumors, and those are not influenced by concurrent administration of progesterone. Because of differences in host reactivity and the histogenesis of the tumors, bioassays of progesterone for co-carcinogenic enhancement of skin carcinogenesis were negative with the C57L as the test strain, just as tests for enhanced pituitary tumor development associated with the feeding of synthetic progestin mixtures were negative with C3H and C57/Black 6 mice (Poel, 1966 and 1967). Paired inbred strains, carefully selected because of differences in their tumor propensities, thus appear essential for long-term bioassays, in lieu of just 1 strain of inbred test animal. Indirectly, these observations indicate also the need for parallel studies with random-bred mice, to enable the scientist to relate the highly refined reactions of the inbred animal to an experimental model more akin to a population-at-large.

The reported findings confirm the preliminary observation (Poel, 1965) that progesterone or the progestational state may provide " co-carcinogenic " enhancement of viral, as well as chemically-induced mammary carcinogenesis. The term " co-carcinogen " was introduced by Shear to designate a non-carcinogenic agent capable of promoting the host's neoplastic response to a carcinogen. As used by Greenstein (1954), co-carcinogenesis designated the effect of a non-carcinogenic agent which, in conjunction with a carcinogen administered at too low a level to produce tumors, caused the production of such tumors. That specific usefulness of the term for designating agents that are not carcinogenic *per se*, but enhance carcinogenesis, has almost been lost for at least two reasons:

(a) The term originated and was used almost exclusively in studies of experimental skin carcinogenesis with crude mixtures (croton oil, creosote oil fractions), or agents (urethane, viruses) that themselves were later found to be carcinogenic (Clayson, 1966; Kawamoto *et al.*, 1958; Poel, 1956; Roe, 1956; Salaman, 1958; Salaman and Roe, 1964; Tannenbaum and Silverstone, 1958; Tannenbaum, 1964; Van Duuren, 1968). Most studies with crude croton oil or croton resin have been complicated by their non-carcinogenic, topically necrotizing components, that distort the patterns of carcinogenesis obtained with purified carcinogens. The isolation of weakly carcinogenic phorbol esters that may be responsible for the co-carcinogenic or carcinogenic effects of crude croton oil mixtures has been reported recently by Hecker (1966) and Van Duuren (1968). In the same vein, however, the term has been used to designate additive or synergistic reactions elicited by two or more agents known to be carcinogenic (Berenblum, 1968; Clayson, 1966; Salaman and Roe, 1964; Kawamoto *et al.*, 1958), a phenomenon designated more recently by the term " syncarcinogenesis ".

(b) The term has been applied to vehicles or solvents such as Tweens and Spans (Setala, 1956; Della Porta *et al.*, 1960), whose solvency for a variety of carcinogens, penetration of tissue, and rates of diffusion or absorption exceed those of distilled water, vegetable oils, toluene, or other solvents used in the early days of experimental carcinogenesis. Extensive use of Tween-60 in control animals, and as a solvent for topical and systemically acting carcinogens in our laboratory gave no evidence of carcinogenic or co-carcinogenic potency for this vehicle (Poel, 1963). The existence of a class of co-carcinogens for the skin thus remains a theoretical or speculative possibility requiring experimental validation. Existence of the phenomenon of co-carcinogenesis, however, apparently may be exemplified by enhancement of mammary carcinogenesis with progesterone, an agent demonstrably negative in all tests to date for carcinogenic potency. In that regard, the findings of Bern, Moore, and others may be interpreted to indicate the need to re-investigate the role of other hormones, to ascertain whether they too may not be acting as co-carcinogens, i.e., non-carcinogenic enhancers of target tissues responding to environmental carcinogens.

SUMMARY

A carcinogenicity bioassay, based upon the differential responsiveness of two inbred strains of mice with contrasting spontaneous tumor proclivities, showed exogenous progesterone to be a potent co-carcinogen for viral or chemical induction of mammary cancer in mice, although non-carcinogenic by itself. The findings emphasize the need to limit the term " co-carcinogen " to non-carcinogenic cofactors, other than solvents, which enhance the neoplastic responsiveness of a host's tissue to a carcinogen.

The experiments reported herein were supported in part by a General Research Support Grant from the National Institutes of Health, FR-05451-07.

I am indebted to Robert Dunson, Maryanne Frickanisce, Mary Harris, and Dolores Stanton for their devoted participation in this study.

### REFERENCES

BERENBLUM, I.—(1968) *Prog. exp. Tumor Res.*, **11**, in press.
BERN, H. A.—(1960) *Science, N.Y.*, **131**, 1039.
BONSER, G. M., DOSSETT, J. A. AND JULL, J. W.—(1961) ' Human and Experimental Breast Cancer ', London (Pitman Med. Publ. Ltd.) p. 130.
BOOT, L. M., MÜHLBOCK, O., ROPCKE, G. AND TENGBERGEN, W. VAN E.—(1962) *Cancer Res.*, **22**, 713.
CANTAROW, A., STASNEY, J. AND PASCHKIS, K. E.—(1948) *Cancer Res.*, **8**, 412.
CLAYSON, D. B.—(1966) *Can. Cancer Conf. of* 1964, **6**, 199.
CUTTS, J. H.—(1966) *Can. Cancer Conf. of* 1964, **6**, 50.
DAO, T. L.—(1964) *Prog. exp. Tumor Res.*, **5**, 157.
DELLA PORTA, G., SHUBIK, P., DAMMERT, K. AND TERRACINI, B.—(1960) *J. natn. Cancer Inst.*, **25**, 607.
DMOCHOWSKI, L.—(1953) *Adv. Cancer Res.*, **1**, 103.
GARDNER, W. U., PFEIFFER, C. A. AND TRENTIN, J. J.—(1959) ' Physiopathology of Cancer ', 2nd edition, New York (Harper) p. 152.
GREENSTEIN, J.—(1954) ' Biochemistry of Cancer ', 2nd edition, New York (Academic Press) p. 76.
HALL, W. T. AND MOORE, D. H.—(1966) *J. natn. Cancer Inst.*, **36**, 181.
HECKER, E.—(1966) Panel on ' Biochem. of Carcinogenesis '. IX International Cancer Congress, Tokyo.
HUGGINS, C., BRIZIERELLI, G. AND SUTTON, H.—(1959) *J. exp. Med.*, **109**, 25.
JULL, J. W.—(1966) *Can. Cancer Conf. of* 1964, **6**, 109.
KAWAMOTO, S., IDA, N., KIRSCHBAUM, A. AND TAYLOR, G.—(1958) *Cancer Res.*, **18**, 725.
KIRSCHBAUM, A.—(1957) *Cancer Res.*, **17**, 432.

MOORE, D. H.—(1963) *Nature, Lond.*, **198**, 429.
MÜHLBOCK, O.—(1956) *Adv. Cancer Res.*, **4**, 371.
NANDI, S.—(1966) *Can. Cancer Conf. of* 1964, **6**, 69.
NOBLE, R. L.—(1964) ' The Hormones ', New York (Academic Press), Vol. 5, p. 559.
POEL, W. E.—(1956) *Science, N.Y.*, **123**, 588; (1963) *J. occup. Med.*, **5**, 22; (1965) *Br. J. Cancer*, **19**, 824; (1966) *Science, N.Y.*, **154**, 402; (1967) *Proc. Am. Ass. Cancer Res.*, **8**, 54.
ROE, F. J. C.—(1956) *Br. J. Cancer*, **10**, 72.
RUSSFIELD, A.—(1966) ' Tumors of Endocrine Glands and Secondary Sex Organs ', P.H.S. Publ. No. 1332 (U.S. Govt Printing Office).
SALAMAN, M. H.—(1958) *Br. med. Bull.*, **14**, 116.
SALAMAN, M. AND ROE, F. J. C.—(1964) *Br. med. Bull.*, **20**, 139.
SETALA, H.—(1956) *Acta path. microbiol. scand.* Suppl., **115**, 1.
SHEAR, M.—(1938) *Am. J. Cancer*, **33**, 499.
SHIMKIN, M.—(1945) ' Mammary Tumors in Mice '. Edited by F. Moulton (AAAS), p. 85.
TANNENBAUM, A.—(1964) *Natn. Cancer Inst.* Monograph No. 14, p. 341; (1964) *Cancer Res.*, **24**, 361.
TANNENBAUM, A. AND SILVERSTONE, H.—(1958) *Cancer Res.*, **18**, 1225.
VAN DUUREN, B.—(1968) *Prog. exp. Tumor Res.*, **11**, in press.